# Genome-wide analysis and molecular dissection of the *SPL* gene family in *Fraxinus mandshurica*

**DOI:** 10.1186/s12870-022-03838-9

**Published:** 2022-09-21

**Authors:** Biying He, Shangzhu Gao, Han Lu, Jialin Yan, Caihua Li, Minghao Ma, Xigang Wang, Xiaohui Chen, Yaguang Zhan, Fansuo Zeng

**Affiliations:** 1 State Key Laboratory of Tree Genetics and Breeding, Northeast Forestry University, Harbin, 150040 China; 2 College of Life Science, Northeast Forestry University, Harbin, 150040 China; 3 Shijiazhuang Academy of Agricultural and Forestry Sciences, Shijiazhuang, 050041 China

**Keywords:** *Fraxinus mandshurica*, *SPL*, Genome-wide, miR156, Abiotic stress

## Abstract

**Background:**

SQUAMOSA promoter binding protein-like (*SPL*) is a unique family of transcription factors in plants, which is engaged in regulating plant growth and development, physiological and biochemical processes. *Fraxinus mandshurica* is an excellent timber species with a wide range of uses in northeastern China and enjoys a high reputation in the international market. *SPL* family analysis has been reported in some plants while *SPL* family analysis of *Fraxinus mandshurica* has not been reported.

**Results:**

We used phylogeny, conserved motifs, gene structure, secondary structure prediction, miR156 binding sites, promoter cis elements and GO annotation to systematically analyze the *FmSPLs* family. This was followed by expression analysis by subcellular localization, expression patterns at various tissue sites, abiotic stress and hormone induction. Because *FmSPL2* is highly expressed in flowers it was selected to describe the *SPL* gene family of *Fraxinus mandshurica* by ectopic expression. Among them, 10 *FmSPL* genes that were highly expressed at different loci were selected for expression analysis under abiotic stress (NaCl and Cold) and hormone induction (IAA and ABA). These 10 *FmSPL* genes showed corresponding trends in response to both abiotic stress and hormone induction. We showed that overexpression of *FmSPL2* in transgenic *Nicotiana tabacum* L. resulted in taller plants, shorter root length, increased root number, rounded leaves, and earlier flowering time.

**Conclusions:**

We identified 36 *SPL* genes, which were classified into seven subfamilies based on sequence analysis. *FmSPL2* was selected for subsequent heterologous expression by analysis of expression patterns in various tissues and under abiotic stress and hormone induction, and significant phenotypic changes were observed in the transgenic *Nicotiana tabacum* L. These results provide insight into the evolutionary origin and biological significance of plant *SPL*. The aim of this study was to lay the foundation for the genetic improvement of *Fraxinus mandshurica* and the subsequent functional analysis of *FmSPL2*.

**Supplementary Information:**

The online version contains supplementary material available at 10.1186/s12870-022-03838-9.

## Introduction

Plants have inherent properties that allow for a variety of physiological, cellular, and molecular responses to be involved throughout development and are susceptible to biotic and abiotic stresses [1]. These developmental processes have been suggested to be regulated by various transcription factors (TFs) [2]. The TF *SPL* is a plant-specific regulatory gene that plays an important role in plant growth and development [3]. The SBP-box genes were first discovered in *Antirrhinum* *majus* and named *AmSBP1* and *AmSBP2* according to their ability to bind to the promoter of the floral meristem identity gene *SQUAMOSA* [4]. The *SPL* genes encode a highly conserved SBP domain containing approximately 76 amino acid residues, including two tandem zinc fingers (Cys-Cys-His-Cys and Cys-Cys-Cys-His), and possess a nuclear localization signal (NLS) at the C-terminus [5]. Based on the important role of the *SPL* gene family in plant floral development, ranging from vegetative to reproductive growth [6], male sterility [7], gibberellin(GA)synthesis [8], and floral morphogenesis [9], researchers have identified *SPL* genes in a growing number of species: 16 *SPL* genes in *Arabidopsis* [10], 28 *SPL* genes in *P. trichocarpa* [11], 21 *SPL* genes in *P. hybrida* [12], and 18 *SPL* genes in *B. luminifera* [13]. Although some similarities are shared among the same clade of *SPL* genes, many of the *SPL* genes from the same clade possess different functions in different plant species [14].

MiR156 targets *SPL* transcription factor gene, which is a main regulator of developmental transition. In addition, in *Arabidopsis thaliana*, *SPL*s interact with the master transcription factor *ABI5* to promote abscisic acid (ABA) signaling [15]. The miR156/*AtSPL2* pathway affects floral organs, silique development and plant fertility, and directly regulates *ASYMMETRIC LEAVES 2 (AS2)* expression [9]. In *Populus*, *SPL* genes play a role in dormancy and flower induction [16]. In *Betula platyphylla*, *BplSPL8* can delay flowering by reducing sensitivity to GA under short-day light and *BplSPL8* controls the number and morphogenesis of leaves, including up-rolling leaves under long-day light and folded leaves mediated by GA under short-day light [16]. In maize, increasing evidence suggests that *ZmSPL* genes play an important roles in regulating maize flowering time, plant/ear height, tilling, leaf angle, tassel and ear architecture, and grain size and shape [17]. In *Lotus japonicus*, miR156-targeted genes, *SPLs* and *WD40* prolong the developmental transition, delay flowering time and enhance shoot branching [14]. In cotton, miR157/*SPL* axis controls floral organ growth and ovule production by regulating MADS-box genes and auxin signal transduction [18].

With the completion of whole genome sequencing of *Fraxinus mandshurica*, the genome wide database can be used for systematic screening, identification and comparative genomics research, providing a rich resource for investigating the biological functions of *SPL* gene family members. *Fraxinus mandshurica* is a kind of dioecious and wind-pollinated tree species belonging to the family *Oleaceae* [19], which is mainly distributed in Northeast China, in addition, there are some small populations in North-west China, the Russian Far East, Japan and North Korea [20]. *Fraxinus mandshurica* has short taproot and well-developed lateral and capillary roots, prefers cold and wet climates, light and fertile soils [21]. Due to its good wood quality and beautiful grain, *Fraxinus mandshurica* is used for making furniture and special construction materials [22]. *Fraxinus mandshurica* is widely used in traditional Chinese medicine (TMC), such as in the TMC formulas for treatment of inflammatory, urinary retention, and fever, especially for the treatment of rheumatism arthritis (RA) in Chinese folk medicine [23]. However, due to long-term overexploitation and extensive deforestation, few mature trees are found to be distributed over large areas [22]. *Fraxinus mandshurica* is now a threatened species and thus declared a national endangered tree species in China [24]. Recent sequencing of the whole genome of *Fraxinus excelsior* [25] and the genome of *Fraxinus mandshurica* [26] has made genome-scale analysis possible. 36 *FmSPL* genes, named *FmSPL1-FmSPL36*, were identified based on the genome, and then the detailed gene structure, phylogenetic relationships and tissue expression profiles under different stress conditions were investigated. We also overexpressed *FmSPL2* in tobacco and preliminarily investigated the function of *FmSPL2* by phenotyping. Our results will provide a basis for further studies on the functional characteristics of the *SPL* gene family members of *Fraxinus mandshurica*.

## Results

### Phylogenetic analysis and classification of *FmSPL* genes

**Fig. 1:**
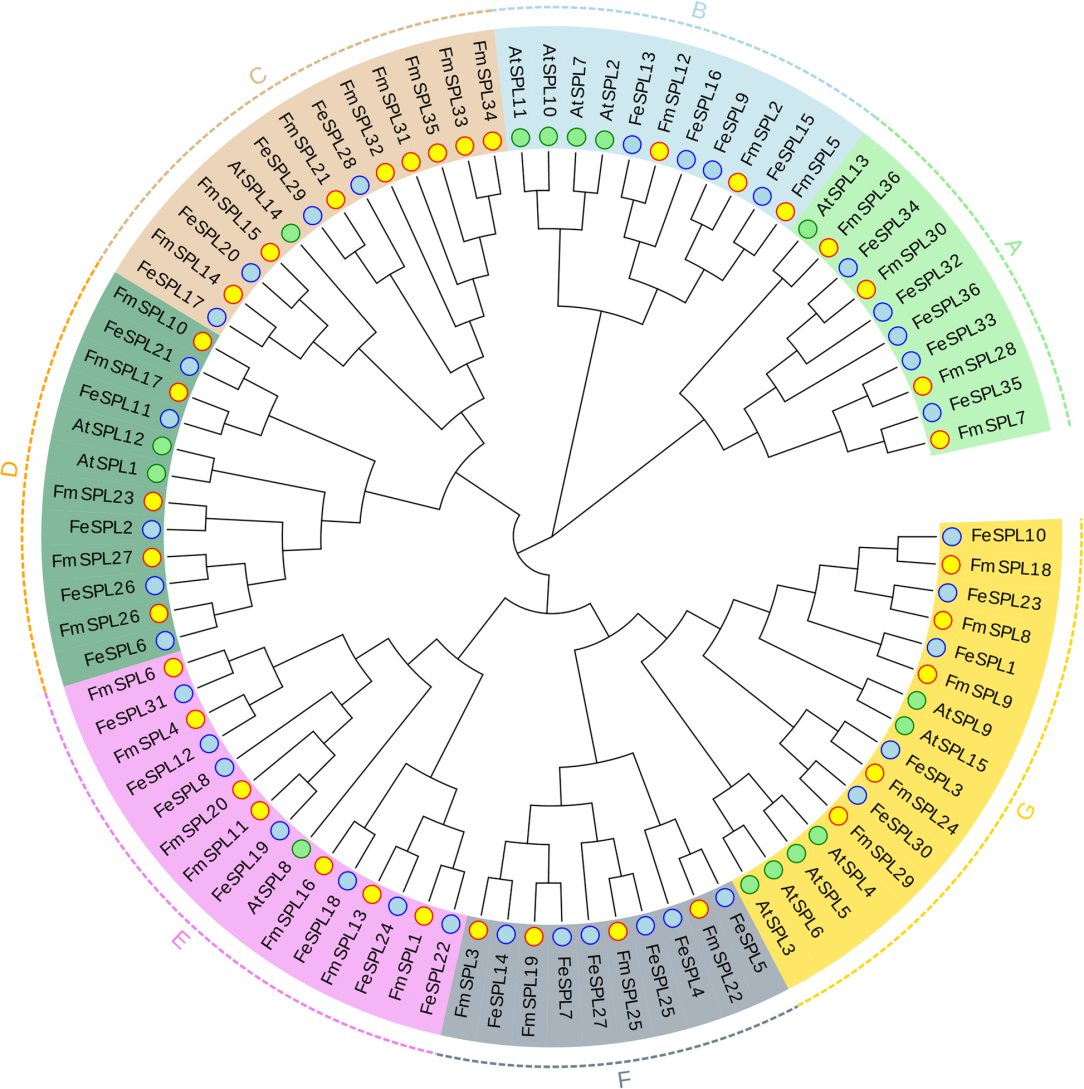
Phylogenetic tree of 87 *SPLs* from three species. The phylogenetic tree was constructed based on the SBP domain using the neighbor-joining (NJ) method with MEGA X. The number on the branch indicates the bootstrap value, and the *SPL* genes in the same species are represented with the same colors: Yellow, *Fraxinus mandshurica*; Blue, *Fraxinus excelsior*; Green, *A. thaliana*

To study the evolutionary relationship of *SPL* genes between *Fraxinus mandshurica* and other plants, we collected 15 *AtSPLs* from *Arabidopsis*, 36 *FmSPLs* from *Fraxinus mandshurica*, and 36 *FeSPLs* from *Fraxinus excelsior* for analyzing a phylogenetic tree. The Neighbor Join (NJ) method was used to construct a rootless phylogenetic tree (Fig. 1). The 87 *FmSPL* genes were divided into seven subfamilies in this phylogenetic tree (A-G). A NJ phylogenetic tree was constructed based on SPL protein sequences from *Fraxinus mandshurica, Fraxinus excelsior* and *A. thaliana* to demonstrate the phylogenetic relationships between Woody and herb plants. The sequences of *A. thaliana* were found to be distributed in A, B, C, D, E and G. However, in F, only sequences of *Fraxinus mandshurica* and *Fraxinus excelsior* were found. In C, *Fraxinus mandshurica* had the highest number of *FmSPL* sequences, while E contained seven *FmSPL* sequences. Moreover, the *SPL* of *Fraxinus mandshurica* and the *SPL* of *Fraxinus excelsior* clustered in one group (Fig. 1) and were more closely related.

### Gene structural characteristics and conserved motif compositions of *FmSPLs* genes

**Fig. 2:**
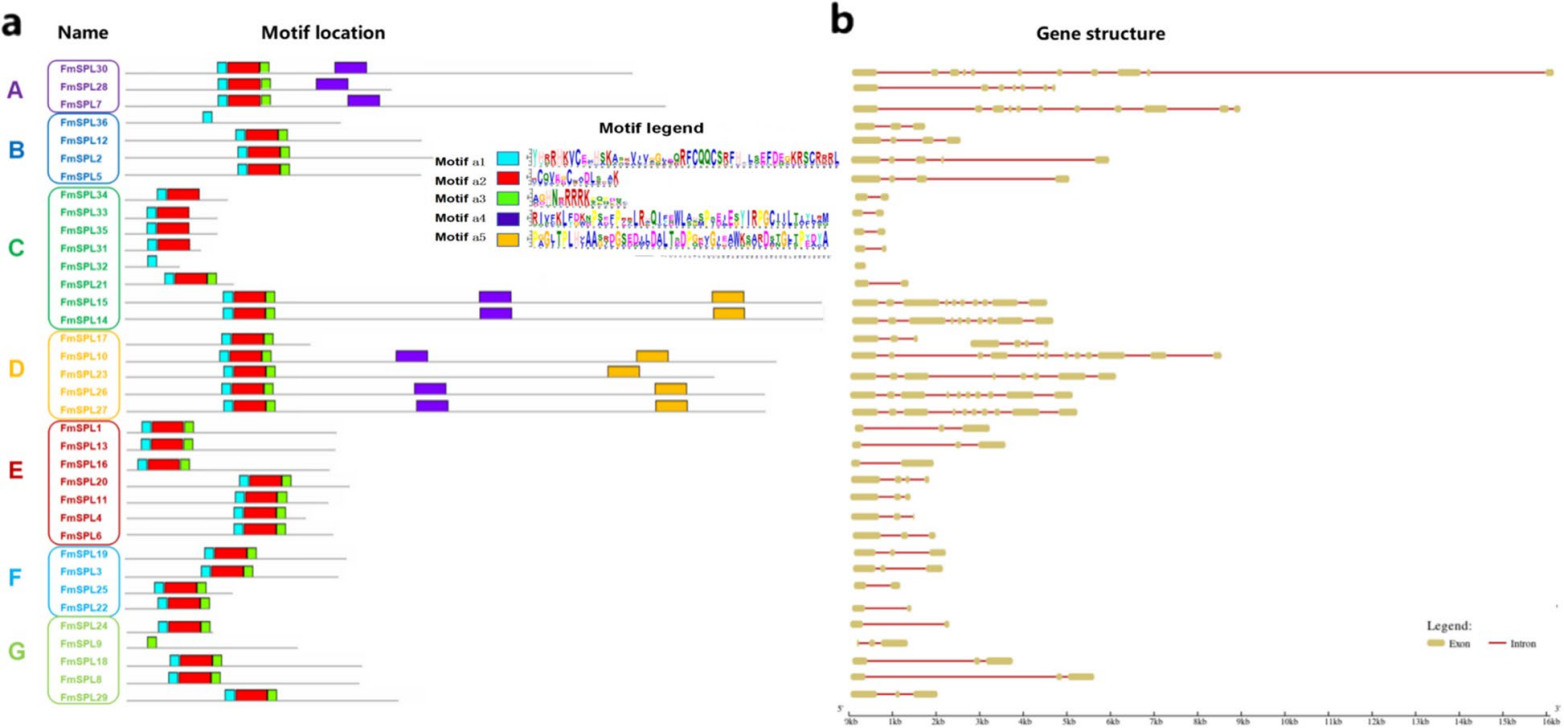
Gene structures and compositions of the conserved protein motifs of the *FmSPL* genes from *Fraxinus mandshurica*. **a** The motif compositions of *FmSPL* genes. The motifs are numbered a1–a5and displayed in different colored boxes. **b** Exon–intron structures of *FmSPL* genes. Yellow boxes indicate exons, and red lines indicate introns

To further examine the structural characteristic of the *FmSPL* genes family, exon–intron distribution and conserved motifs were analyzed (Fig. 2). The length of motifs ranged from 15 to 50 amino acids and the number of motifs varied between 1 and 5 in each FmSPL protein. Most of the FmSPLs in group D shared motifs a4 and a5, but FmSPLs in the group A were mainly motif a4. Most of the FmSPLs in group B, C, E, F and G contained a1, a2 and a3. Among 36 FmSPLs sequences, only 6 sequences contained a5 motifs, which were in group C and group D. FmSPL9 contains only one a3 motif. Most FmSPL members within the same clade, particularly the most closely related members, generally shared common motif compositions (Fig. 2a). In order to study the structural composition of *FmSPL* genes, we studied the exon and intron in detail including their amount and distribution (Fig. 2b). The coding regions of many genes are interrupted by introns in a variety of genetic systems and organisms [27]. In general, the closest members from the same subgroups had a similar exon/intron structure with regard to intron number, intron phase, and exon length. Gene structure analysis of FmSPLs showed that the exon/intron organization varied in number and length (number of introns ranged from 1 to 10). Exon–intron structural diversity is considered to play an important role in the evolution of *FmSPL* genes. The divergence of gene structure (the exon–intron and conserved motifs diversity) provides the potential insights of gene function of evolution.

**Fig. 3:**
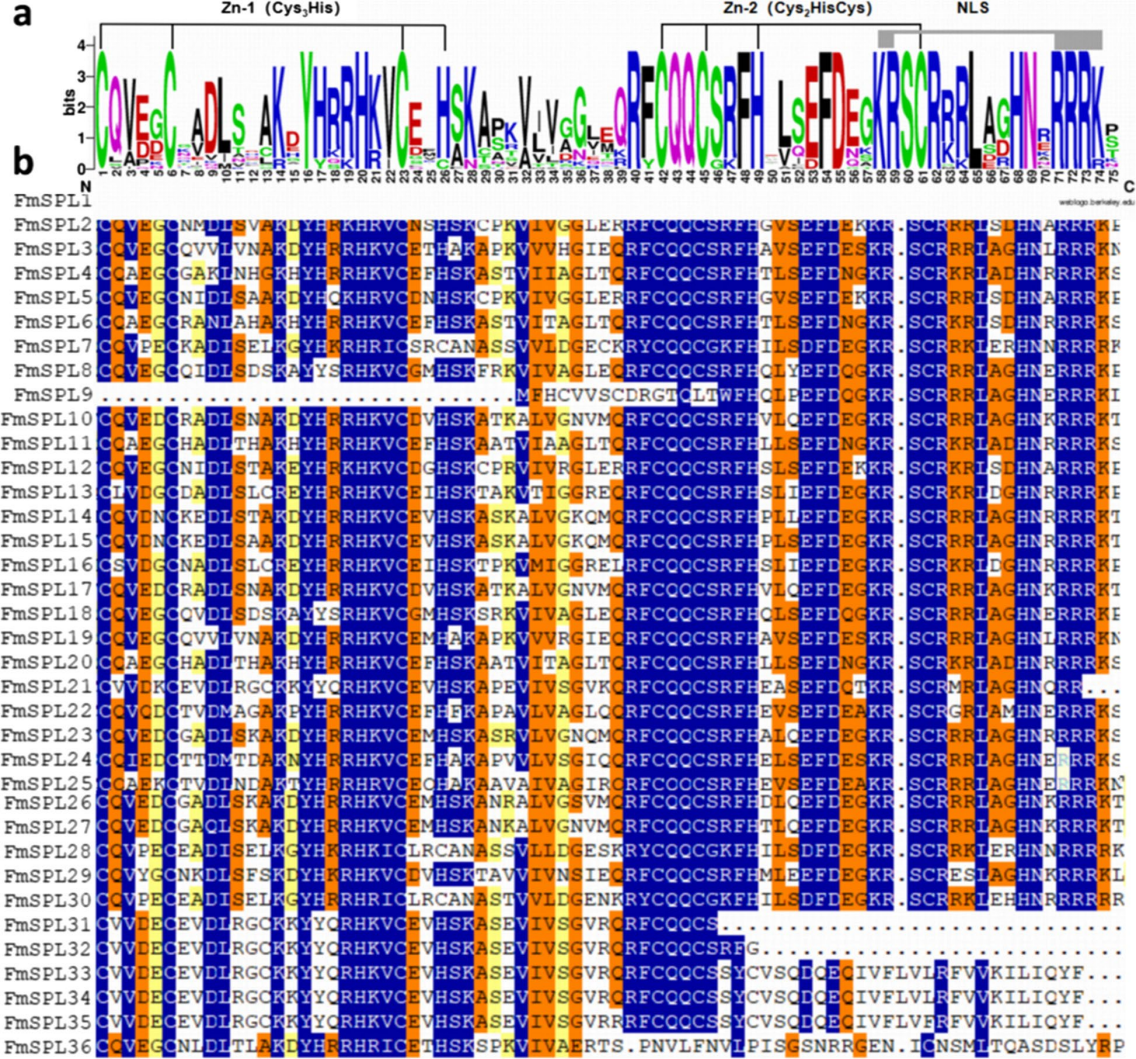
Sequence alignment and conserved motif of the FmSPLs. **a** Sequence conserved motif of the SBP domain from the FmSPLs. **b** Multiple alignments of the FmSPLs

High similarity of the SBP domains, especially several extremely conserved positions, was observed in all FmSPLs. All of the SBP domains in FmSPL proteins contain two zinc finger-like structures (Zn-1 and Zn-2), and all the FmSPLs contain a conserved nuclear location signal (NLS) in the C-terminus of SBP domains, which was partly overlapped with the Zn-2 motif (Fig. 3a). Only the protein sequences of the highly conserved SBP domains were used for phylogenetic analysis, as alignment of the full-length protein sequences revealed that only the SBP domains were conserved (Fig. 3b).

### Pre-miRNA secondary structure prediction

**Fig. 4:**
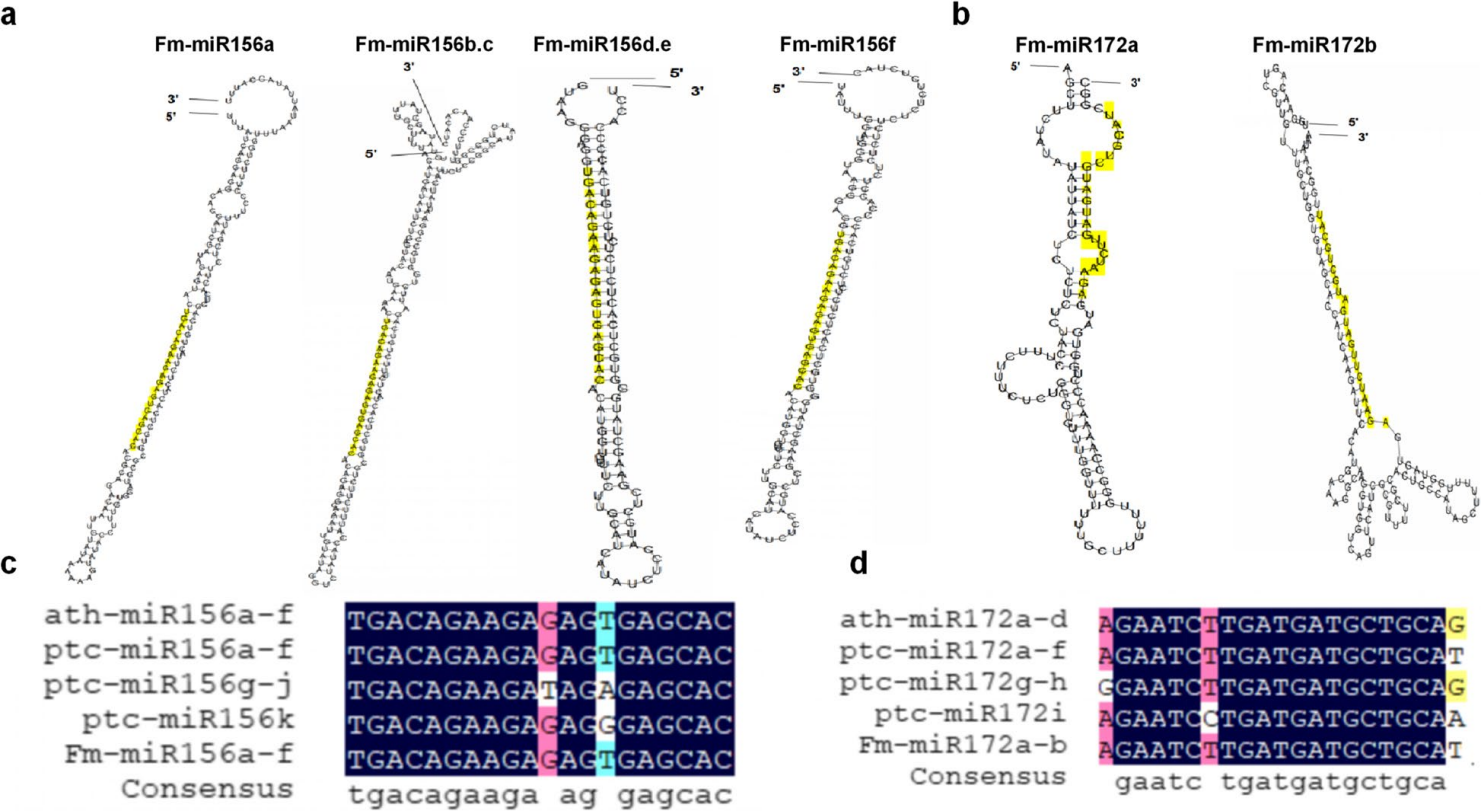
Predict the stem-loop structure of Pre-miRNA of Fm-miR156 (**a**) and Fm-miR172 (**b**) Use RNAfold to predict the secondary structure. The predicted mature miRNA sequence is shown in yellow. Alignment of miR156 (**c**) and miR172 (**d**) mature sequences

To identify the conserved miRNAs in *Fraxinus mandshurica*, the miRNAs sequences were searched against known miRNAs in miRBase by similarity with a maximum of two mismatches and without gaps (Fig. 4). The putative precursor sequences of these predicted miRNAs were further analyzed using RNAfold software to confirm their stem-loop structures (Fig. 4a, b). Generally, the miRNA process needs the Drosha to produce the pre-miRNA and then processed to mature miRNA by Dicer [28]. Expression analysis is used to determine which miRNAs are processed into mature sequences in vivo. Different miRNA members in the same plant species are encoded by different gene loci, of which the precursors vary (Fig. 4a.b), but the mature sequences were identical or highly similar (Fig. 4c.d). As can be seen from the Fig. 4, the miR156a-f sequences of *Arabidopsis*, *Populus tomentosa* and *Fraxinus mandshurica* are very conservative, while FmmiR156g-j and FmmiR156k of *Fraxinus mandshurica* have two and one different nucleotides compared with other miR156, respectively. Similarly, the mature sequence of miR172 is also very conservative. Compared with *Arabidopsis* miR172, *Populus tomentosa* and *Fraxinus mandshurica* have only one or two different nucleotides.

### Analysis of miR156 target sites in 14 *FmSPL* genes

**Fig. 5:**
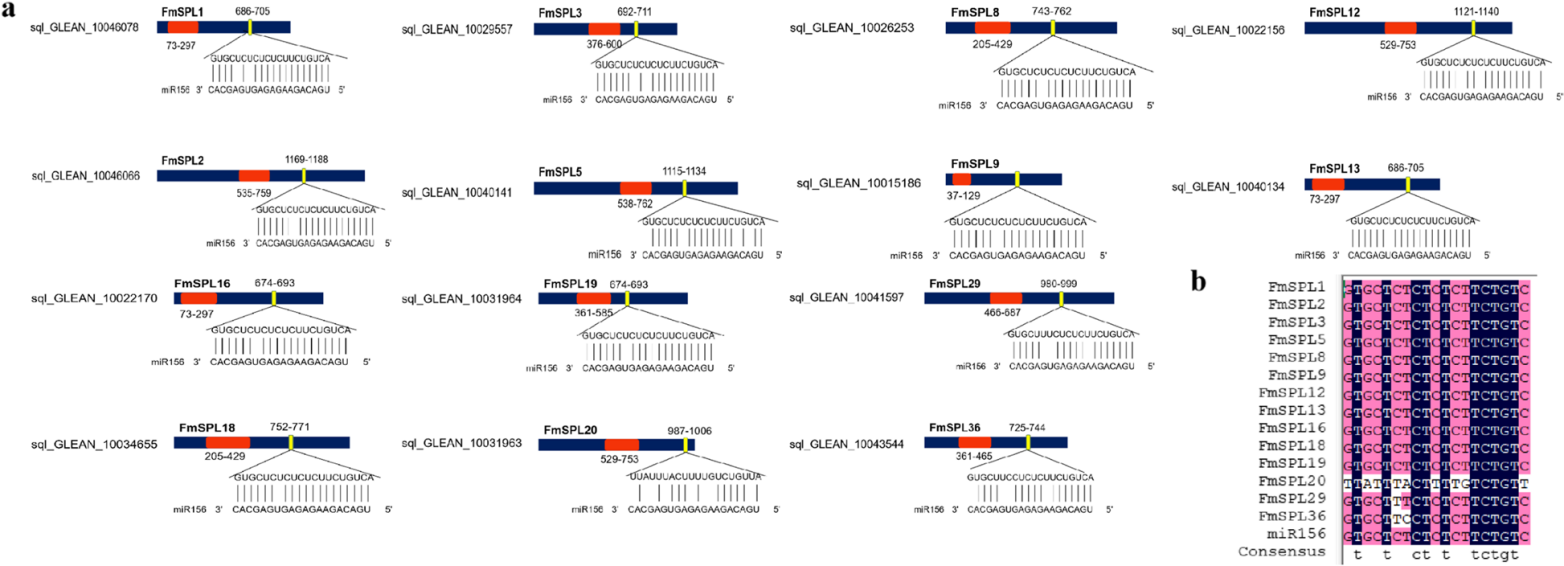
FmmiR156 sequence and binding sites of FmmiR156 in *FmSPL* genes. **a** Diagram of *FmSPL* sequences targeted by miR156. The blue boxes represent CDS regions; the orange boxes represent the SBP domains; and the yellow boxes represent miR156 target sites of *FmSPL* transcripts. In the expanded regions, the sequence direction of *FmSPL* is from 5′ to 3′ and the miR156 sequence direction is from 3′ to 5′. **b** Sequence alignments of FmmiR156 with their complementary sequences of *FmSPLs*

Because of the important functions of the miR156/*SPLs* pathway in plant growth, it was important to explore *SPLs* in *Fraxinus mandshurica* that can be regulated by miR156. To identify the *FmSPLs* targeted by miR156, we searched the coding regions of all *FmSPLs* for targets of *Fraxinus mandshurica* miR156 using the psRNATarget online prediction tool with default parameters. Fourteen (*FmSPL1*, *FmSPL2*, *FmSPL3*, *FmSPL5*, *FmSPL8*, *FmSPL9*, *FmSPL12*, *FmSPL13*, *FmSPL16*, *FmSPL18*, *FmSPL19*, *FmSPL20*, *FmSPL29* and *FmSPL36*) of *FmSPLs* have target site of miR156 and they are predicted to be the potential targets of miR156 (Fig. 5a). All potential FmmiR156 targets were located downstream of the SBP domain in the coding region and matched exactly to the FmmiR156 sequence. *FmSPL29*, *FmSPL36*, and *FmSPL20* may have FmmiR156 targets in the coding region, but there were several mismatch sites (Fig. 5b), suggesting that miR156 may specifically target these genes in *Fraxinus mandshurica*. These obtained FmmiR156 targets were involved in a wide range of biological process and played important regulatory roles in plant growth and development.

### *Cis*-elements in the promoter regions of *FmSPL* genes

**Fig. 6:**
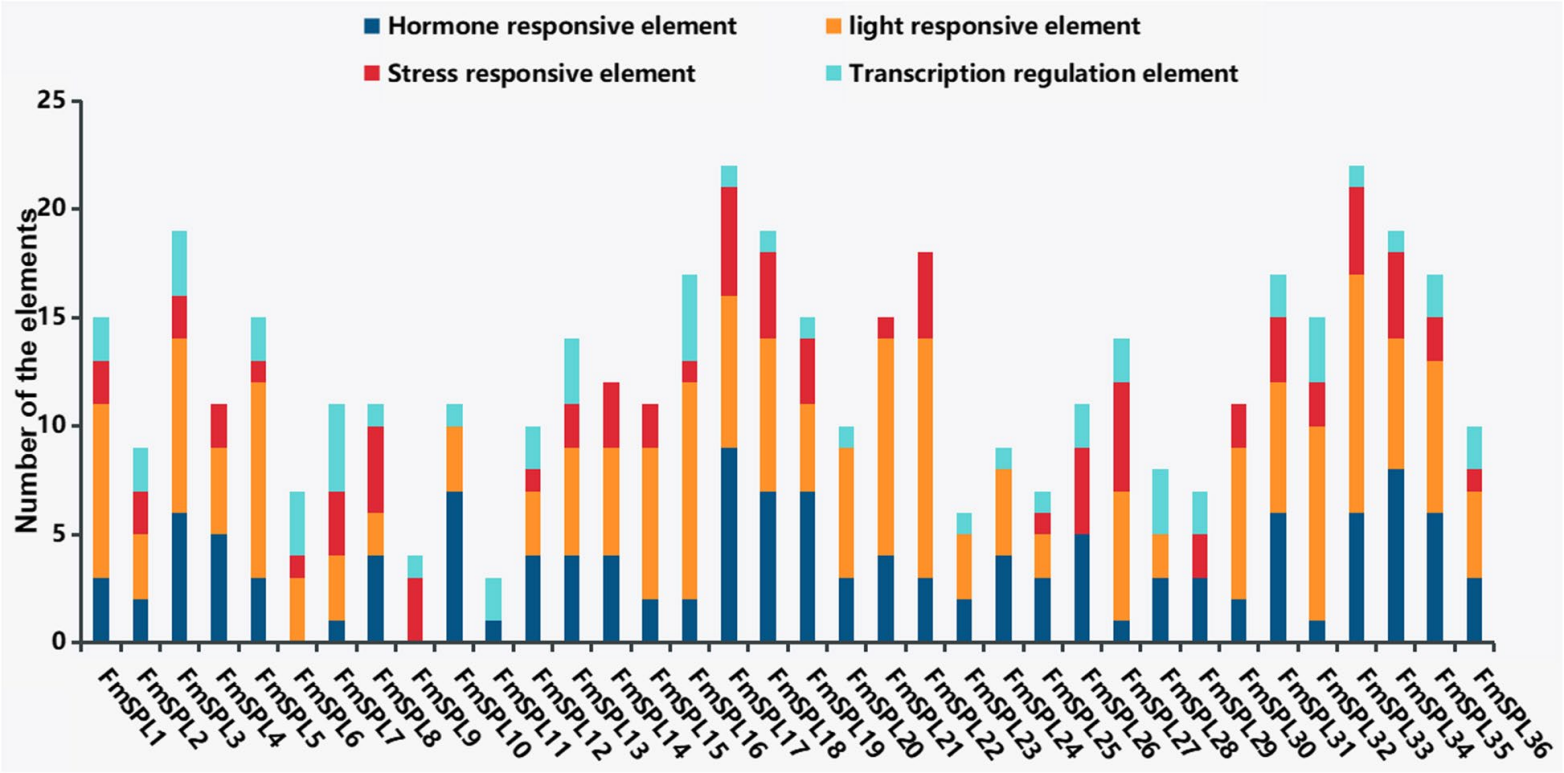
Cis-element regions of 36 FmSPL promoters were analyzed by PlantCARE. The elements associated with specific functions are indicated by different colors for each gene

To understand the transcriptional regulation and potential function of the *FmSPL* genes, we analyzed the *cis*-regulatory elements in the promoter sequences using PlantCARE software. According to their putative functions, these elements were categorized into four classes (Fig. 6). The results showed that light responsive elements were the largest group and were present in almost all of the *FmSPL* gene promoter regions, except in *FmSPL9*, *FmSPL11*, *FmSPL26* and *FmSPL29*. The second major group was the hormone response elements including ABA, methyl jasmonate (JA), salicylic acid (SA), GA and auxin (IAA), which is present in the promoter regions of almost all *FmSPL* genes except *FmSPL6* and *FmSPL9*. Then, except for *FmSPL10*, *FmSPL11*, *FmSPL20*, *FmSPL23*, *FmSPL24* and *FmSPL28*, there are stress response elements in the *FmSPL* genes promoter region. Most gene promoter regions can bind MYB transcription factors to regulate transcription. This suggested that *FmSPLs* could be regulated by various environmental and developmental changes, which implied that *FmSPLs* play an important roles in physiological processes and developmental events.

### Gene Ontology (GO) annotation

**Fig. 7:**
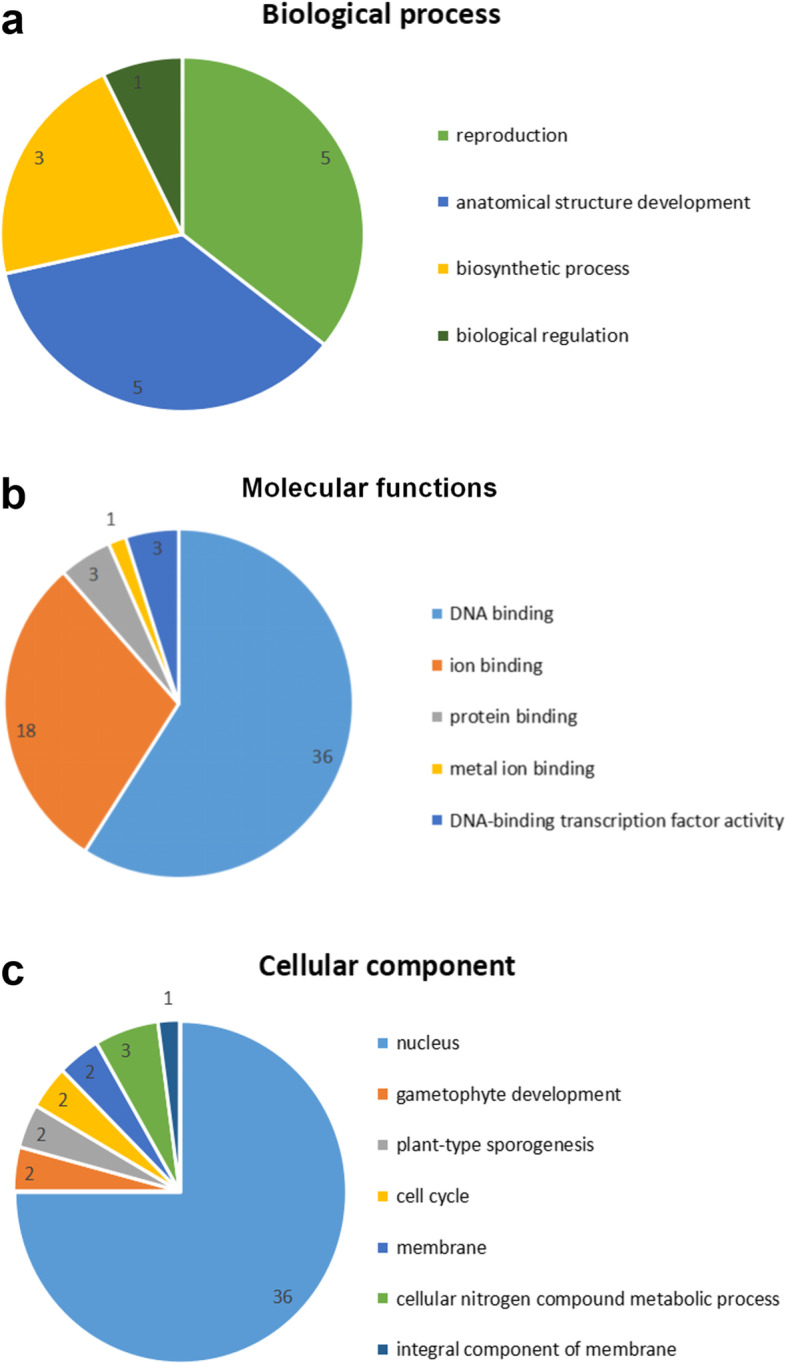
The gene ontology analysis of *FmSPL* gene family. The GO analysis of *FmSPL* genes predicted for their involvements in (**a**) biological process, (**b**) molecular functions, and **c** cellular components

GO analysis was performed to determine the function of these genes in biological processes (Fig. 7). The genes were categorized according to the annotation of GO, and the number of every category is displayed based on biological process, cellular components, and molecular functions. Cellular process and molecular functions had the largest number of *FmSPL* genes, followed by regulation of biological process. Among them, 5 *FmSPL* genes are involved in biological reproduction, 2 *FmSPL* genes are involved in gametophyte regulation, and 3 *FmSPL* genes are involved in cellular nitrogen compound metabolism. All *FmSPL* genes are involved in DNA binding and nuclear development.

### Subcellular localization of *FmSPL* proteins

**Fig. 8:**
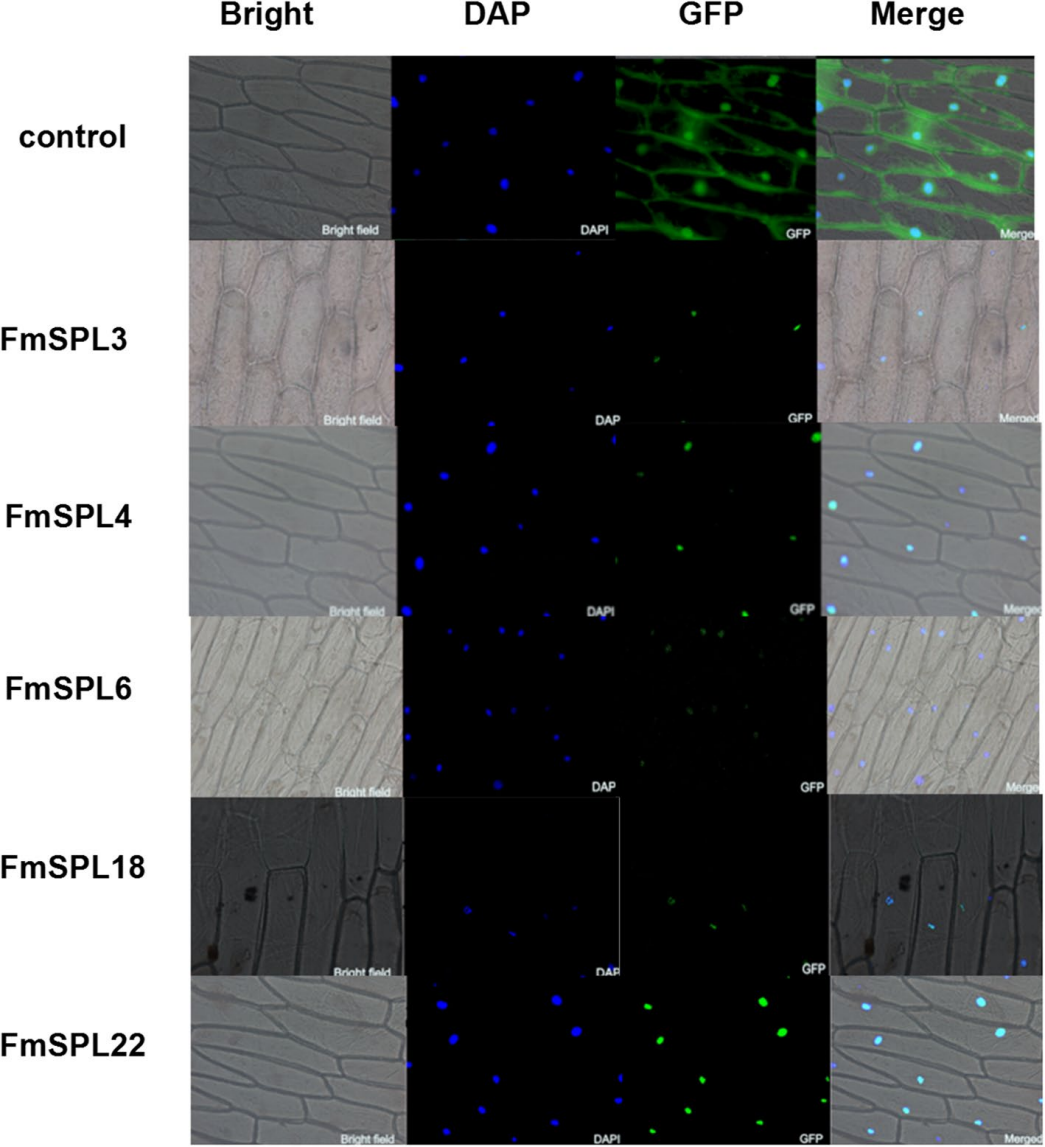
Subcellular localization of FmSPLs. The constructed expression vector transiently infects onion epidermal cells, stained with DAPI and observed under a fluorescence microscope

We transformed 35S: FmSPLs-GFP into onion epidermal cells by Agrobacterium-mediated transient infusion, using 35S: GFP as a control. The results showed that the control GFP fluorescence was spread throughout the entire cellular structures, including the nucleus, cytomembrane and cytoplasm (Fig. 8). Green signal represents GFP fluorescence. DAPI fluorescent signal (blue) indicates the presence of nucleus. The merged section shows the combined fluorescent signal from the DAPI-stained nucleus (blue) and the FmSPL protein (green). The results of protein subcellular localization showed that FmSPLs (FmSPL3, FmSPL4, FmSPL6, FmSPL18, FmSPL22) proteins were located in the nucleus.

### Digital expression profiling of *FmSPL* genes in various tissues of *Fraxinus mandshurica*

**Fig. 9:**
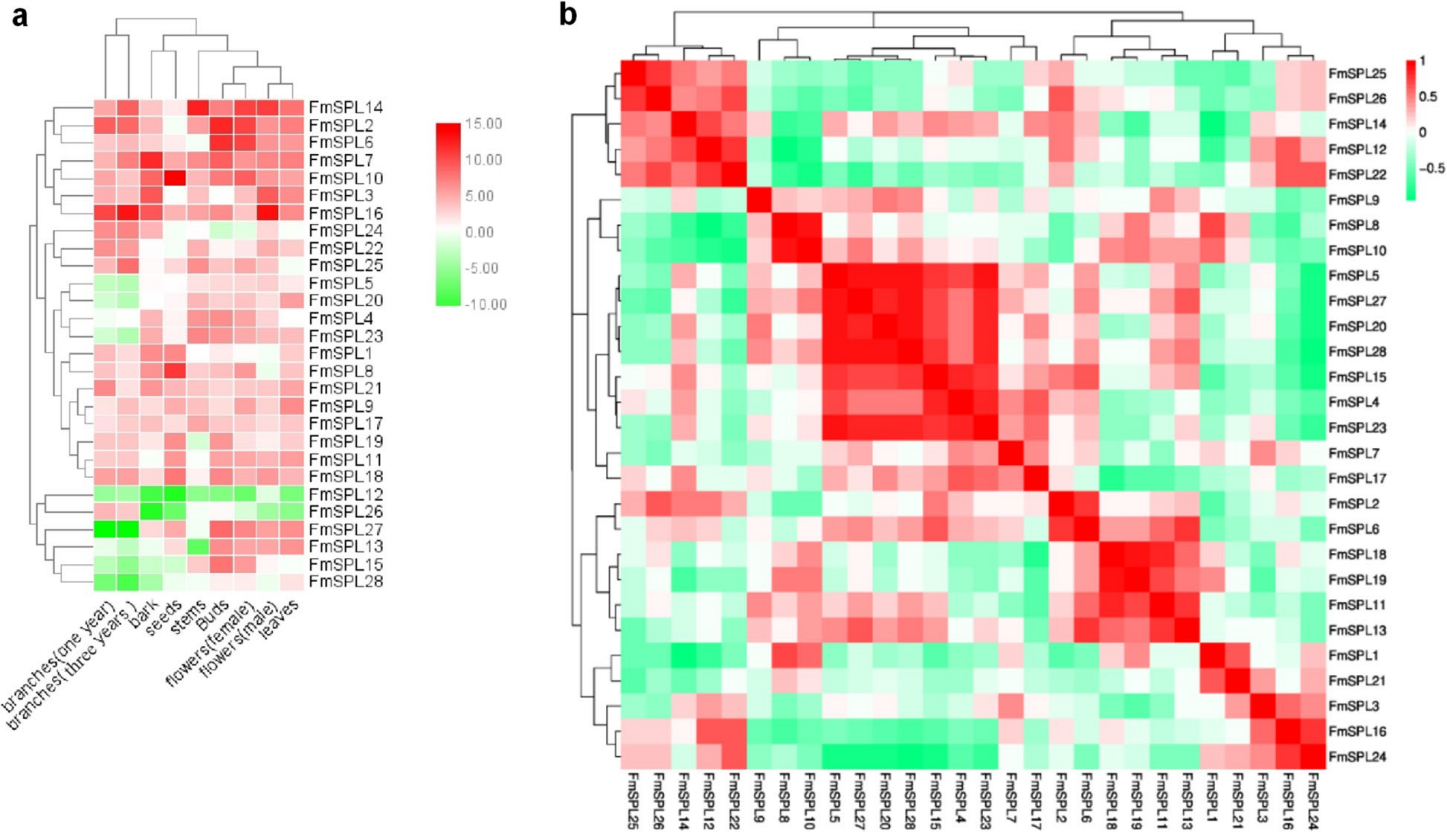
Expression patterns of *FmSPL* genes. **a** Cluster analysis of gene expression patterns of *Fraxinus mandshurica SPL* family. **b** Correlation analysis of gene expression patterns of *Fraxinus mandshurica SPL* family

In order to gain insight into the assumed functions of these genes in *Fraxinus mandshurica*, 28 *FmSPL* genes were selected to study their temporal and spatial expression patterns by RT-qPCR analysis. As shown in Fig. 9a, *FmSPL2*, *FmSPL6* and *FmSPL14* showed highest expression in the buds and female flowers, but *FmSPL1* relatively low expression. *FmSPL16* was highly expressed and *FmSPL12* was lowly expressed in male flowers and bark. *FmSPL10* and *FmSPL8* was highly expressed in seeds, however, the expression level of *FmSPL3* is low. *FmSPL14* was highly expressed in stems. The spatial variations in the expression level of *FmSPL* genes in different tissues revealed that they may participate in different growth and development processes in *Fraxinus mandshurica*. We further analyzed and found that genes with similar expression patterns showed positive correlation in expression pattern correlation analysis. *FmSPL25, FmSPL26, FmSPL14, FmSPL12* and *FmSPL22* were correlated with each other. *FmSPL5, FmSPL27, FmSPL20, FmSPL28, FmSPL15, FmSPL4* and *FmSPL23* had some correlation with each other. *FmSPL2, FmSPL6, FmSPL18, FmSPL19, FmSPL11* and *FmSPL23* had some correlation with each other (Fig. 9b).

### Expression pattern of *FmSPLs* at phenological states

**Fig. 10:**
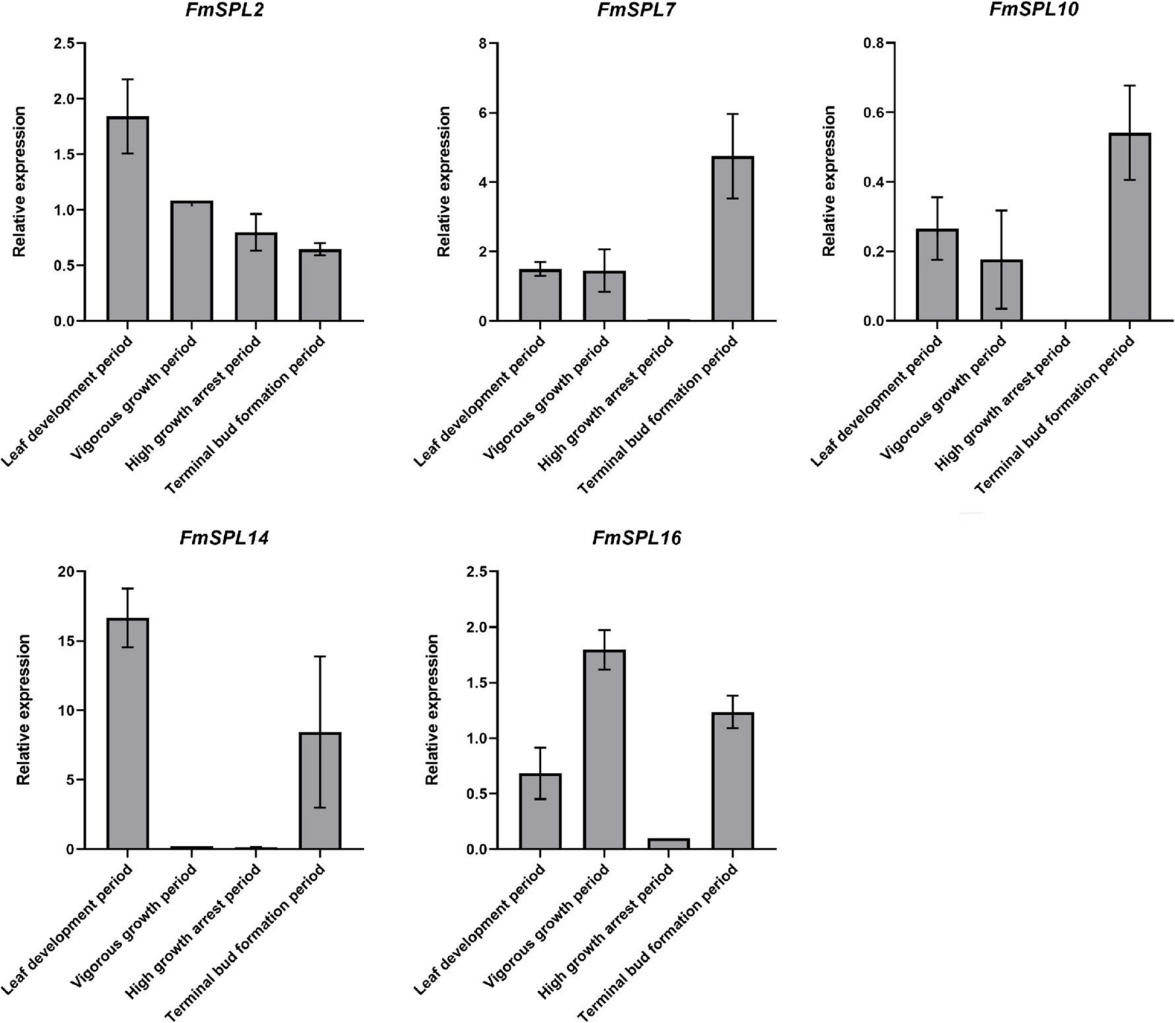
Expression pattern of *FmSPL* gene in *Fraxinus mandshurica* leaves at phenological states

The genes with high expression levels at each site were selected to further reveal the role of the gene in the regulation of growth and development at different phenological states (Fig. 10). Fluorescence quantitative PCR was performed on the samples of *Fraxinus mandshurica* leaves at different phenological states. The results showed that the expression of *FmSPL2* gradually decreased over time, with the highest expression in leaf development period, which was 3.74 times higher than that in terminal bud formation period. The expression patterns of *FmSPL7* and *FmSPL10* were similar, and both had the highest expression in terminal bud formation period, being 91-fold and 125-fold higher than that in high growth arrest period, respectively. *FmSPL14* expression was highest in leaf development period, decreased in vigorous growth and high growth arrest period, and increased in terminal bud formation period, with the expression in leaf development period being 2.25 times higher than that in terminal bud formation period. The expression of *FmSPL16* was elevated from the leaf development period to the vigorous growth period. In contrast, the expression decreased significantly in the high growth arrest period and recovered in the terminal bud formation period. Therefore, the expression of *FmSPL16* was highest in vigorous growth period and lowest in high growth arrest period, with a 19-fold difference between the two. The differences in *FmSPL* gene expression patterns under different phenological states of *Fraxinus mandshurica* further suggest that different *FmSPL* genes are involved in different stages of growth and development of *Fraxinus mandshurica*.

### Expression patterns of *FmSPL* genes under different abiotic stresses and hormone induction

**Fig. 11:**
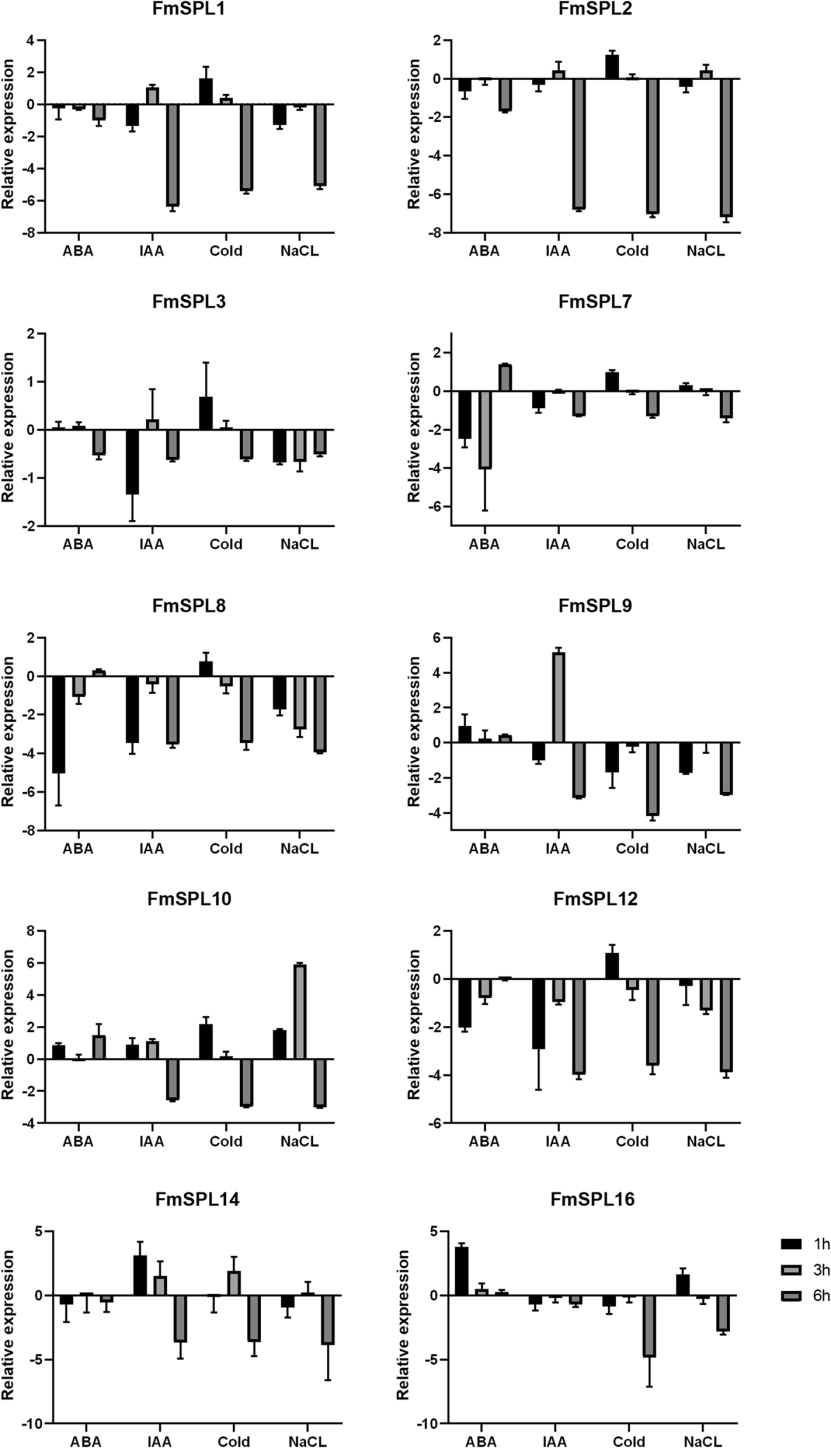
Quantitative RT-PCR analysis of 10 *FmSPL* genes. Relative expression levels of 10 genes under 4 different treatments (ABA, IAA, NaCl and Cold)

*SPL* genes are involved in abiotic stress, plant hormone regulation and other signaling processes. We selected 10 genes (*FmSPL1, FmSPL2, FmSPL3, FmSPL7, FmSPL8, FmSPL9, FmSPL10, FmSPL12*) that were highly expressed at different sites and at different times, respectively, and subjected to abiotic stress (cold, NaCl) and hormonal treatments (ABA, IAA) for three time periods (1 h, 3 h, 6 h) (Fig. 11). Under ABA treatment, *FmSPL7* and *FmSPL10* were first down-regulated in expression and gradually up-regulated when the induction time was extended. Similarly, under ABA induction, *FmSPL8* and *FmSPL12* were significantly down-regulated at 1 h of treatment, and the down-regulated expression gradually diminished when the time was prolonged, and had down-regulated expression at 6 h. The trend for most genes under IAA treatment was that expression was down-regulated at 1 h, up-regulated by 3 h and down-regulated by 6 h. Under cold treatment, the trend was consistent for most genes, with expression levels gradually down-regulated with increasing time. Under NaCl stress, most genes were down-regulated, but there was an up-regulated expression trend in *FmSPL2*, *FmSPL7*, *FmSPL10* and *FmSPL16* at the corresponding time points. Among the 10 selected *FmSPLs* genes, *FmSPL9* was most significantly up-regulated at 3 h of IAA induction, and *FmSPL10* was most significantly up-regulated at 3 h of NaCl stress. The results suggested that *FmSPLs* might have potential roles in stress adaptation and various hormone signal responsiveness.

### Ectopic Expression of *FmSPL2* in *Nicotiana tabacum L*

**Fig. 12:**
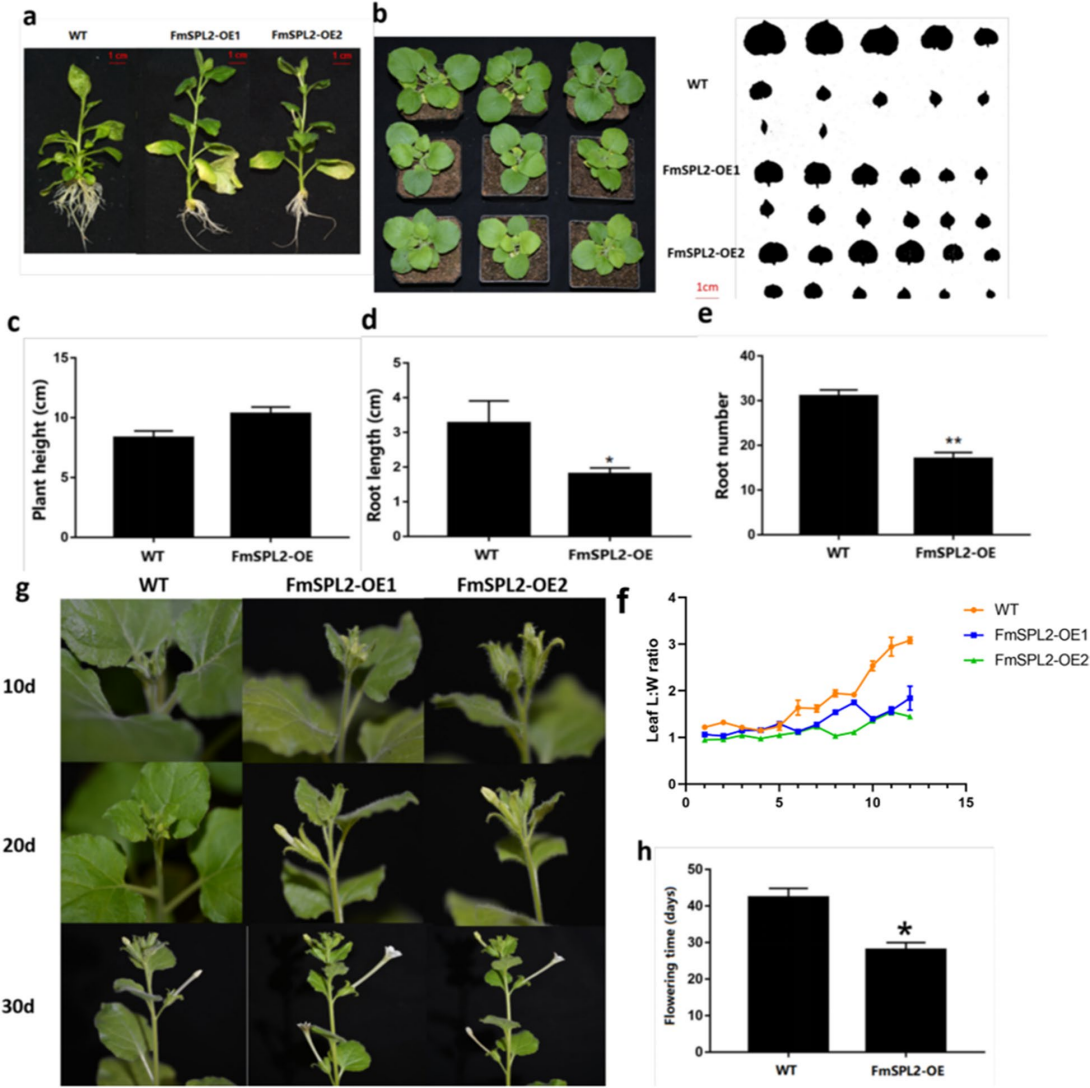
The phenotype of transgenic tobacco with FmSPL2. **a** Wild-type and transgenic tobacco phenotypes. **b** Wild-type and transgenic tobacco leaves. **c** Plant height. d Root length. **e** Root number. **f** Leaf aspect ratio of wild-type and transgenic tobacco. **g** Phenotype of wild-type and transgenic tobacco flowers at different times (10d, 20d, 30d). **h** Flowering time. Single or double stars at the top of each column in the figure indicate a significant difference among genotypes at 0.01 < *p* ≤ 0.05 and *p* < 0.01, respectively, based on the student’s t-test

Since *FmSPL2* is not only highly expressed in flowers and shoots but also has a significant response in abiotic and hormonal regulation, we further investigated the function of *FmSPL2* through transgenic *Nicotiana tabacum* (Fig. 12). Two independent transgenic lines were selected for phenotypic observation. The phenotype of the transgenic *Nicotiana tabacum* showed (Fig. 12a), the transgenic plant was taller than the wild type (Fig. 12c), the root length (Fig. 12d) was shorter than the wild type, and the number of roots was less (Fig. 12e). The leaves overexpressing *FmSPL2* were smaller than those of the wild type (Fig. 12b). As seen from the aspect ratio (Fig. 12f), there was no significant difference between mature leaves, while the aspect ratio of young leaves of the wild type was larger than that of the transgenic type. It can be indicated that the wild type juvenile leaves are narrow and long, while the *FmSPL2* transgenic plants have round juvenile leaves. The transgenic *FmSPL2* plants had a shorter flowering time (Fig. 12g), from which it can be seen that at 10 days the transgenic plants were in the budding period while the wild-type plants had no obvious flower buds. 20 days later, it can be seen that the wild-type plants were in the budding period while the transgenic plants had reached the initial flowering period, and 30 days later, it can be seen that the transgenic plants were in the full flowering period while the wild-type plants had just reached the first After 30 days, it can be seen that the transgenic plants are at full flowering while the wild-type plants are just at the initial flowering. As a result, the flowering time of transgenic *Nicotiana tabacum* was reduced by about 10 days compared to the wild type (Fig. 12h). It indicates that *FmSPL2* is involved in the whole growth and development process of plants.

## Discussion

### Characteristics of *SPL* family genes in *Fraxinus mandshurica*

*SPL* genes, a major family of plant specific transcription factors, play an important role in regulating several important and diverse biological processes in plants, including secondary metabolite metabolism, leaf, flower, flowering time, plant architecture, juvenile to adult transition, and fruit development etc. [29]. The above biological processes are important for the growth and development of *Fraxinus mandshurica* and its application in production. Since its discovery in *A. majus*, homologous *SPL* genes have also been found in *algae*, *mosses*, *Arabidopsis* and crops. However, less research has been reported on *SPL* genes in perennial woody plants [11, 30]. Most *SPL* genes expanded in a species-specific manner after the split of monocotyledons from dicotyledon, and gene duplication plays important role in *SPL* gene expansion in *P. trichocarpa* [11, 31]. *Fraxinus mandshurica* is an important and timber species whose *SPL* gene has not been reported.

In this experiment, phylogenetic analysis, gene structure, conserved motifs, GO annotation, miR156 analysis as well as target gene prediction and promoter region analysis were performed for the *SPL* family of *Fraxinus mandshurica*. Here, 36 *FmSPL* genes from *Fraxinus mandshurica* were screened using a genome-level database constructed from the Pfam database (PF03110) and the *FmSPL2* gene clones were validated.

Comparative analysis of the *FmSPLs* revealed many conserved sequence features. For instance, all of the deduced proteins contain the highly conserved SBP domain with about 78 amino acid residues. Based on the neighbor-joining (NJ) phylogenetic tree constructed using MEGA 10. 87 SPL proteins from *Fraxinus mandshurica*, *Fraxinus excelsior* and *Arabidopsis* were found to cluster into 7 groups. Most groups included at least one *AtSPL*, and only one group had no *AtSPL*, but *FeSPL* was distributed in each group, indicating a closer relationship between *FeSPL* and *FmSPL* than the evolution of *AtSPL*. The number of introns, intron phase and conserved pattern of *FmSPLs* within a group were similar. These results suggest that there is consistency among *FmSPLs*. The mature sequences of miR156 and miR172 are very conserved, but the precursor sequences are different, and multiple different precursor sequences can end up synthesizing the same mature sequence in vivo. Target prediction indicated that the genes regulated by miR156 in *FmSPL* are in groups A, B, E, F and G of the phylogenetic tree, while *FmSPL* in groups C and D is not regulated by miR156. The complementary sites of miR156 locate in the coding region of *SPLs*. It implies that the post-transcriptional regulation mediated by miR156 is conserved in plants. Post-transcriptional regulation of miR156/*SPL* is important for the function of *SPLs* [32–40]. In this experiment, some phylogenetic and predictive analyses were performed to gain a preliminary understanding of the development and potential functions of *FmSPL* and to lay the foundation for subsequent in-depth studies on the *FmSPL* gene in terms of phenotype, stress and hormone induction.

### Expression patterns and potential functions of *FmSPL* genes

At present, there was few functional data of *SPL* genes available in *Fraxinus mandshurica*. Therefore, our study investigates the potential functions of *Fraxinus mandshurica SPL*. Cis-elements in promoter regions are closely correlated with *SPL* gene function. It was found that many cis-elements were associated with light, hormone, and stress responsiveness, which was similar with *Betula luminifera* [13]. Among the cis-acting elements, tissue-specific elements such as endosperm and phloem expression were also detected, as well as transcriptional regulatory components, and MYB binding sites were involved in drought induction. And through GO annotations, it is found that each *FmSPL* gene has molecular functions and cellular components, which can indicate that *FmSPLs* participate in more biological processes and have certain biological functions.

In *A. thaliana* and *tree peony*, most *SPL* genes are expressed in various tissues and organs and at different developmental stages [29, 41]. Most of *FmSPL* genes show high expression levels in flower buds and young fruit in *Prunus mume* [11]. We used RT-qPCR to determine the expression of *FmSPL* gene during growth and development. *FmSPL8* and *FmSPL10* have higher expression in seeds, and *FmSPL2* and *FmSPL6* have higher expression in buds and flowers. *FmSPL8* and *FmSPL10* can be predicted to have a role in fruit development and seed maturation. And *FmSPL2* and *FmSPL6* have a function in flower development and may regulate flowering time. Interestingly, *FmSPLs* are highly expressed in flowers, and miR156 is involved in regulating the function of *SPL* genes. miR156/*SPL* can regulate the phase transition of plant morphology, the formation of floral organs, flowering time, gibberellin signaling, response to stress, and disease resistance produced by cleavage of *SPL* transcription factors [42, 43]. Among them, flowering is a physiological development process that transforms plants from vegetative growth to reproductive growth, which indicates that miR156/*SPL* participates in the regulation of plant age. Exogenous sugars trigger the *Arabidopsis* juvenile-to-adult phase transition via a miR156A/*SPL* module [44]. *Fraxinus mandshurica* is a perennial woody plant. These strongly suggest that *FmSPL2* may be responsible for the reproductive stage transition of *Fraxinus mandshurica*. This was confirmed by overexpression of *Fraxinus mandshurica SPL2* into *Nicotiana tabacum* which revealed earlier flowering phenotype (Fig. 12g-h). The role of *SPLs* genes in the control of lateral root development has been previously demonstrated [45]. The results of this paper suggest that *FmSPL2* may also play a role in root development (Fig. 12). Together, these results in our study uncover the functional roles of *Fraxinus mandshurica SPL* genes and provide important candidates for further functional characterization with the aim of genetic improvement of growth and development traits. Since *FmSPLs* were differentially expressed in different parts of *Fraxinus mandshurica*, it was found that the expression of *FmSPLs* was influenced by the phenological states. Among them, *FmSPL2* and *FmSPL14* were highly expressed at flowering stage (April–May), and SPL2 was found in our overexpressing SPL plants to cause earlier flowering in tobacco (Fig. 12g), which is consistent with our speculation, and in *Jatropha curcas*, *JcSPL3* overexpression caused earlier flowering in the plants [35]. *FmSPL10* was highly expressed at the fruiting stage (August–September), it could indicate that *FmSPLs* are involved in the whole developmental process of *Fraxinus mandshurica*. In addition, we analyzed the expression level of *FmSPL* genes induced by ABA, IAA, NaCl and low temperature (4 °C) to predict potential effects. The stress-related phytohormone ABA inhibits seed germination and seedling growth to adapt various environmental challenges [15]. It was shown that protein kinases SnRK2s interact with and phosphorylate *SPL9*, which is essential for its role in the activation of ABA responses [46]. Previous studies have shown that *SPL* is involved in the response to abiotic stresses, such as salt and drought [6, 47]. The expression of most *FmSPLs* genes is differentially regulated in a given stress response and hormone induction, which strongly suggests that they may be important adversity adaptation and hormone induction genes. Our results indicate that the *SPL* gene may play an important regulatory role in the induction of ABA, IAA, NaCl and low temperature in *Fraxinus mandshurica*.

These results indicated that the overexpression of *FmSPLs* can change the flowering time of plants, and can exhibit responsive effects under stress treatment and hormone induction, we’ll do more research on stress response and the function of its *SPL* gene under hormone induction will follow in detail.

## Conclusion

We identified 36 *FmSPL* genes in this work. Because they have a conserved SBP-box structural domain, they belong to the *SPL* gene family. Phylogenetic analysis showed that these 36 *FmSPLs* were clustered into seven groups. Meanwhile, most of the *FmSPLs* in the same group showed a high degree of consistency in gene structure and conserved motif composition. Fourteen of the *FmSPL* genes had miR156 target sites, and the target sites were all in the CDS region. Promoter cis-element analysis of the *FmSPLs* indicated that they play important roles throughout development and physiology. Analysis of gene expression patterns further indicated that *SPLs* have the potential to regulate flowering, lateral bud and seed development, as well as the juvenile to adult stage transition in *Fraxinus mandshurica*. Among them, overexpression of *FmSPL2* in *Nicotiana tabacum* was selected and found to alter leaf size, root development, and early flowering phenotypes. These results provide insights into the evolutionary origin and biological significance of plant *SPLs*. They provide a solid foundation for subsequent detailed studies on the functions of *FmSPLs* genes and genetic improvement of *Fraxinus mandshurica*.

## Methods

### Plant materials

Male flowers, female flowers, buds of female plants, branches, different parts of the bark and leaves of *Fraxinus mandschurica* were collected from May to August 2018 from the experimental forestry farm of Northeast Forestry University (Harbin, China). *Fraxinus mandshurica* material was selected for different periods of leaf development period (Mid-May), vigorous growth period (Mid-June), high growth arrest period (Mid-July), and terminal bud formation period (Mid-August). *Nicotiana tabacum* cultivar was used for this study.

### Analysis of plant *SPL* family

The nucleotide and amino acid sequence information of *Arabidopsis AtSPLs* were obtained from the *Arabidopsis* Information Resource (TAIR, http://www.arabidopsis.org/). The relevant sequence information of *Fraxinus excelsior* comes from (http://www.ashgenome.org/JBrowse). *Fraxinus mandshurica* sequence information was based on the transcriptome [48] and genome and genome [49] of our laboratory. The SBP-domain profile (PF03110) downloaded from the Pfam database (http://pfam.xfam.org/, Pfam 30.0) was used as the query sequence against the downloaded CDS sequences of *Fraxinus mandschurica* using the HMMER algorithm. The sequence information of the identified *FmSPL* gene is shown in Additional file 6. After removing short and low-quality readings, 36 *FmSPL* and 36 *FeSPL* sequences were obtained. A bootstrapped neighbor-joining tree containing all *SPL* sequences was constructed by using MEGA 10. Sequence logos were generated using WebLogo (http://weblogo.berkeley.edu/logo.cgi**).** The sequences used in the phylogenetic tree are in Additional file 4.

### Exon/intron structures and conserved motif analysis

The exon/intron structure of each *FmSPLs* gene was generated by using the online Gene Structure Display Server 2.0 (http://gsds.cbi.pku.edu.cn/). To investigate conserved motifs in more detail, we determined the number and type of conserved motif for all *FmSPLs* genes. To identify shared motifs and structural divergences among the predicted full-length FmSPLs proteins, the MEME online tool (http://meme.nbcr.net/meme/intro.html). The maximum number of motifs was set at 5, and the optimum motif widths were set at between six and 200 residues.

### Promoter *cis*-element analysis

The Tbtools gene family analysis software was used to determine the promoter (2.0 kb upstream of the coding sequence) and the repetitive sequence analysis of the *FmSPLs* coding sequence. The *cis*-acting regulatory elements in the *FmSPLs* promoter region were analyzed using the PLACE database (http://www.dna.affrc.go.jp/PLACE/). The putative cis-acting elements enriched in the promoter of *FmSPLs* are shown in Additional file 3.

### Gene Ontology (GO) annotation

The functional annotation of *FmSPL* genes was performed using Blast2GO (version 5.2.5) as follows: the full-length protein or nucleic acid sequences were aligned and mapped using UniProtKB/Swiss-Prot (swissprot_v5). Annotation Contlguration as follow: E-value-hit-filte of 1.0E-6, Annotation cutoff: 55, and GO weight: 5, the output date was used for annotating into three GO categories (biological processes, molecular functions, and cellular components).

### Secondary structure prediction

Prediction of secondary structures of the pre-miRNA alleles performed by the RNAfold software (http://rna.tbi.univie.ac.at//cgi-bin/RNAWebSuite/RNAfold.cgi). The MiR156 and miR172 precursor sequences are listed in additional file 2.

### Prediction of *FmSPL* stargeted by miR156

The sequences of the mature miRNAs were obtained from miRBase (http://www.mirbase.org/). *FmSPL* genes targeted by miR156 were predicted by searching the coding sequence regions of 14 *FmSPLs* for complementary sequences using the online psRNATarget server (http://plantgrn.noble.org/psRNATarget/home).

### Subcellular localization of FmSPL proteins

To determine the subcellular localization, full length of coding sequences without stop codon of *FmSPL* genes were fused into pCAMBIA-1303 vector. The expression vector was then transformed into *Agrobacterium tumefaciens* strain *GV3101*. The pCAMBIA-1303 fusion protein signal was localized to the nucleus after being transiently expressed in onion epidermal cells.

### Plant stress treatment and hormone induction

*Fraxinus mandshurica* seeds were germinated and grown on soil in a growth room for about a month. Plant material was grown under greenhouse conditions (16-h light/8-h dark, 56% humidity, 23℃) for the duration of all experiments. To check the expression level of *FmSPLs* under phytohormone treatments and Abiotic stress, the seedlings at four-leaf stage were subjected to low temperature (4℃), NaCl (200 mmol/L), ABA (100 μmol/L) and IAA (100 μmol/L), respectively, each with three replicates. Samples were collected at 1, 3 and 6 h and immediately stored at –80 °C.

### Quantitative RT PCR (RT-qPCR)

Total RNA was extracted using the CTAB method [50]. RNA concentration was measured with NanoDrop, and first-strand cDNA was synthesized using a reverse transcription kit (Takara, Dalian, China). cDNA was diluted 10 times and used as the template. The data were normalized using the reference gene *Fraxinus mandshurica* α-tublin [1, 51], Gene-specific primers for RT-qPCR analysis were listed in Additional file 1. For fluorescence qPCR, a SYBR Green (TOYOBO) kit was used. PCR was performed in a 20 μL reaction volume containing 6.4 μL of sterile distilled water, 10 μL of THUNDERBIRD SYBR qPCR Mix, 0.6 μL each of upstream and downstream primers, 0.4 μL of 50 × ROX reference dye, and 2 μL of cDNA template. Fluorescence qPCR was performed on the Applied Biosystems 7500 Real-Time PCR System [1]. The reaction conditions were as follows: 95 °C for 30 s, 40 cycles of 95 °C for 5 s and 60 °C for 34 s, 95 °C for 15 s, 60 °C for 1 min, and 95 °C for 15 s. All reactions were repeated three times. The y = log^2ΔCT(control)^-log^2ΔCT(treatment)^ algorithm was chosen for the expression pattern of the *FmSPLs* gene under different abiotic stresses and hormone induction. The y = 2^−ΔΔCT^ algorithm was chosen for the expression pattern of *FmSPLs* in the physical state. All data were processed with SPSS 22.

### Genetically Modified *Nicotiana tabacum L*

The full-length CDS sequence of *FmSPL2* was cloned into binary expression vector pCAMBIA-1303 and transferred into the Agrobacterium strain *GV3101*. *Nicotiana tabacum* L. was transformed using the Leaf Disk Method. Plants were grown in growth chamber under long-day conditions. For leaf shape analysis, fully expanded leaves were removed, attached to cardboard with double-sided tape and flattened with transparent tape, and then scanned in a digital scanner. Regeneration process of *FmSPL2* transgenic Nicotiana tabacum L. in Additional file 5. Identification of transgenic positive plants by DNA and cDNA levels was carried out by Minghao Ma. T1 seeds were harvested and selected on MS medium with kanamycin 10 mg/L.

## Supplementary Information

**Additional file 1.**

**Additional file 2.**

**Additional file 3.**

**Additional file 4.**

**Additional file 5.**

**Additional file 6.**

## Data Availability

The datasets used and analyzed during the current study are available in the manuscript and its additional files.

## Abbreviations

NLS: Nuclear localization signal
SBP: SQUAMOSA-promoter binding protein
PCR: Polymerase chain reaction
RT-qPCR: Quantitative real-time reverse transcription PCR
GFP: Green florescence protein
DAPI: 4',6-Diamidino-2-phenylindole
