# Supplementary material for: Genome-wide analysis and molecular dissection of the SPL gene family in Fraxinus mandshurica
Source: BMC Plant Biol. 2022 Sep 21;22:451. doi: 10.1186/s12870-022-03838-9 (PMC9490987; doi:10.1186/s12870-022-03838-9)
Supplement: Supplementary file 1 — Additional file 1. [file 12870_2022_3838_MOESM1_ESM.pdf]

Table S1 Primers used in this study.

Primer name	The sequence (5’-3’)
TU	F-primer:AGGACGCTGCCAACAACTTT
	R-primer:TTGAGGGGAAGGGTAAATAGTG
FmSPL1	F-primer:ATCAAAAGGGGCATAG
	R-primer:GCAATCGCAACCATAGAAA
FmSPL2	F-primer:TAGCAAGAACATTGAAAGC
	R-primer:ATAGAGGTGGATGAAGTAGG
FmSPL3	F-primer:TCACAGTCCCACTTCTATT
	R-primer:TTGCCTTTCTTGGTCAT
FmSPL4	F-primer:ATGTTTCCGTGGTCATTTCCCGTAT
	R-primer:CATTGTGTCTCATTCGCTCTTCCTA
FmSPL5	F-primer:TGAAATCATCTACTCCAAAC
	R-primer:AAGTCCTCCTACAATAACCT
FmSPL6	F-primer:GCATCATCGTCGTTTTCT
	R-primer: ATCTCACAGACTTTATGCC
FmSPL7	F-primer:CTATCTGGAAGAGGCACAAT
	R-primer:AACCGATGAAACGCTAA
FmSPL8	F-primer:ATGGTGGACTATGAATGG
	R-primer:AAGTTATGAGTGGCGTATGG
FmSPL9	F-primer:TAGAGGCTTTGTGATGGA
	R-primer:AATAACTTGTTCGGGTCG
FmSPL10	F-primer:ACTTCGTTATGTTCGGTTCA
	R-primer:CAATGTTCTTGCTAAAATCTCC
FmSPL11	F-primer:TGAAATCATCTACTCCAAAC
	R-primer:ACTGTTGACAAAACCGAC
FmSPL12	F-primer:AATCTCTTCTATGGTCAAGCAGTAT
	R-primer:CATTTCCAGTCATTCAAGTCC
FmSPL13	F-primer:AGAGTTTGACGAAGGAAAGAGAAGC
	R-primer:TTGGATACTTTGGTGAGTGCTTTGC
FmSPL14	F-primer:TCCAGGAACCAAGATACACT
	R-primer:ACTAGATGATGCTCCACGAA
FmSPL15	F-primer:ATGTCTTGGGATTGGGATA
	R-primer:AATGGATACGGGCTTCTT
FmSPL16	F-primer:GTTTCATTCCTTGATAGAGT
	R-primer:AATTCGTGTACCTTGCTG
FmSPL17	F-primer:GCAGTTGTTTCCTGTTGA
	R-primer:ATAACCCTCCTTTTCTCC
FmSPL18	F-primer:GTCAAGTGGAAGGGTGTCAGGTAGA
	R-primer:AGAAGTCCATCACGAAACCTCCATT
FmSPL19	F-primer:AGCGATAGAAGTAGGGAA
	R-primer:AAGCAAGTCAAATGTGGG
FmSPL20	F-primer:ATGGTGGACTATGAATGG
	R-primer:ATTAGTCAAATGGGTGCT

FmSPL21	F-primer:AAATGGCAGAAGAGGATG
	R-primer:TTGACCCCTGAAACTATG
FmSPL22	F-primer:AAGACGAAGATGGGGAAGATGAAGA
	R-primer:GTGCCTGCGATGGTATGGTTTAGC
FmSPL23	F-primer:GCTGACAAAGGATGAGAA
	R-primer:GACTTGACTGATGCGATT
FmSPL24	F-primer:GGAAATGAGCAAAGCAGA
	R-primer:GGAGCCTTAGCATGAAAT
FmSPL25	F-primer:TTACCATCGACGACATAG
	R-primer:AACTTCTTTTCGCTTCAT
FmSPL26	F-primer:ACGGGGATAACAACAACT
	R-primer:TGACAAACTGCACGACTT
FmSPL27	F-primer:TGGAAAACAACCAAGTGA
	R-primer:TGATACAACCAGGTCTAATG
FmSPL28	F-primer:TAAAGGCAGTTTTGAGATG
	R-primer:CCAGATATTTGTAAGGGTG
