# Supplementary figures and images for: Genome-wide analysis and molecular dissection of the SPL gene family in Fraxinus mandshurica
Source: BMC Plant Biol. 2022 Sep 21;22:451. doi: 10.1186/s12870-022-03838-9 (PMC9490987; doi:10.1186/s12870-022-03838-9)

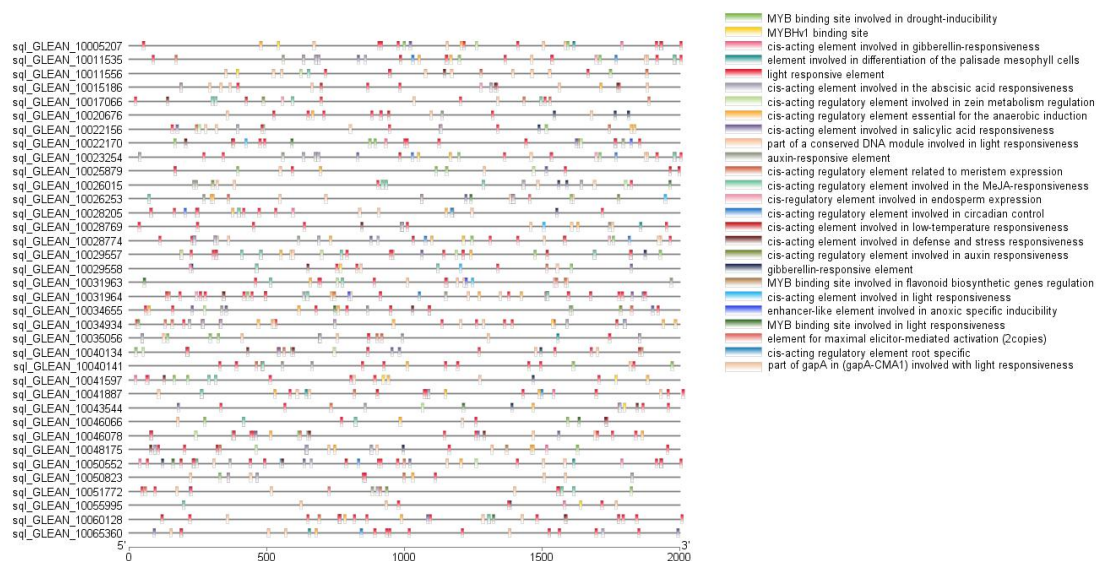

**Fig S1** Putative cis-acting elements enriched in promoters of *FmSPLs*.

Supplement: Supplementary file 3 — Additional file 3. [file 12870_2022_3838_MOESM3_ESM.pdf]
