# Supplementary material for: Genome-wide analysis and molecular dissection of the SPL gene family in Fraxinus mandshurica
Source: BMC Plant Biol. 2022 Sep 21;22:451. doi: 10.1186/s12870-022-03838-9 (PMC9490987; doi:10.1186/s12870-022-03838-9)
Supplement: Supplementary file 5 — Additional file 5. [file 12870_2022_3838_MOESM5_ESM.pdf]

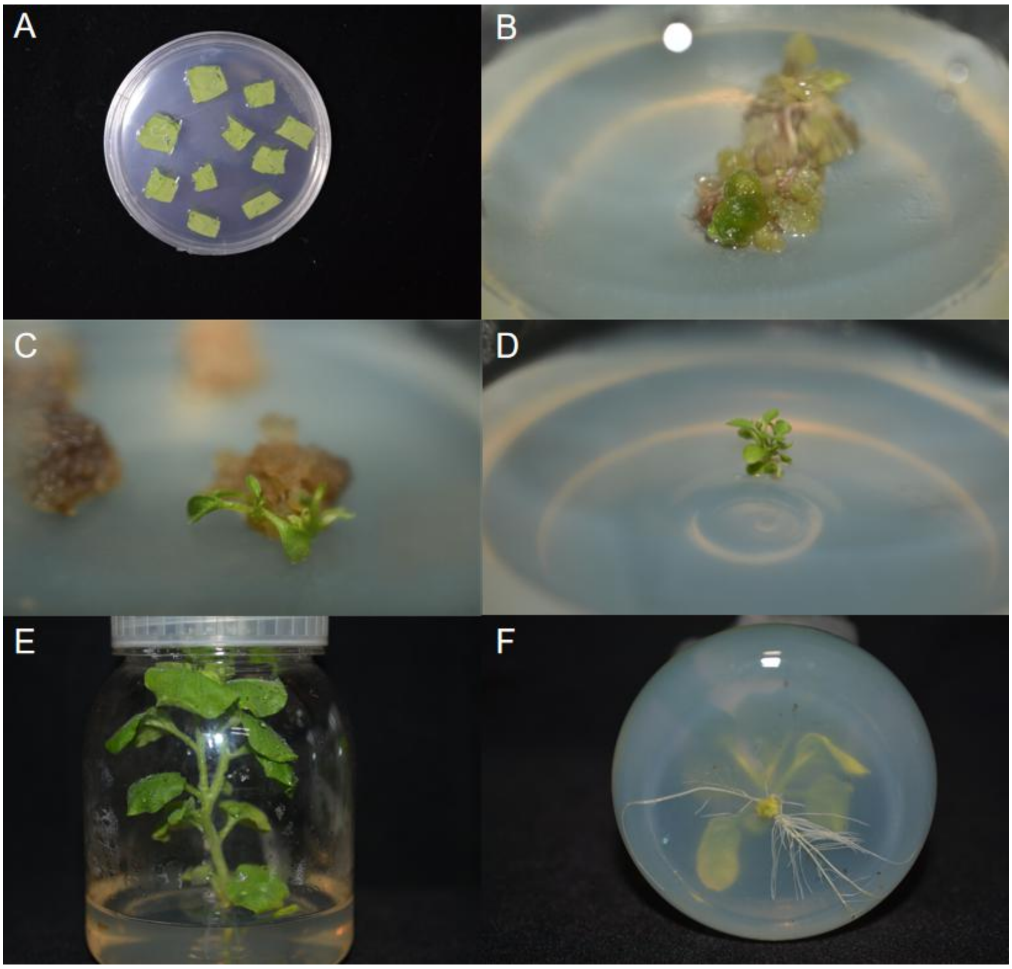

Fig S2 Regeneration process of *FmSPL2* transgenic *Nicotiana tabacum L*. **A** *Nicotiana tabacum L* histopathic seedlings grown to 4-5 leaves for infestation. **B** *Nicotiana tabacum L* leaves forming resistant healing tissues. **C** Germination of resistant healings after 1-4 weeks of induction in germination medium. **D-E** Different periods (8-10 weeks) for the growth of *Nicotiana tabacum L* roots in rooting selection medium when the buds are to be grown to 2-3 cm thick and transferred to rooting medium for strengthening.
